# Reproducible differentiation and characterization of neurons from mouse embryonic stem cells

**DOI:** 10.1016/j.mex.2020.101073

**Published:** 2020-09-22

**Authors:** Sonal Saxena, Sumana Choudhury, K. Naga Mohan

**Affiliations:** Department of Biological Sciences, Birla Institute of Technology and Science Pilani, Hyderabad Campus, Jawahar Nagar, Hyderabad 500 078, India

**Keywords:** Mouse embryonic stem cells, Neuronal differentiation, Cell-based models, Neurological disorders

## Abstract

Investigation on the effects of disease-associated mutations on neurodevelopment is an essential approach to understand the molecular basis of neurological disorders and can be achieved by generating suitable animal models. However, some of the mutations preclude development of animal models, leaving cell-based models as the only options. Mouse embryonic stem cells (mESCs) are attractive because of the well-established technologies for introducing disease-associated mutations and the feasibility of investigating the abnormalities during different stages of neurogenesis. Importantly, such transgenic mESCs enable large-scale screening and identification of the most promising small molecules and/or drug candidates before undertaking expensive animal studies. Although neuronal differentiation from mESCs is one of the earliest methods to be developed, we observed that the published as well as publicly available methods did not yield neurons consistently. Here, we describe a 16-day differentiation protocol that consistently induced differentiation of mESCs into neurons. This step-wise protocol enables monitoring of the neuronal differentiation process at different stages as well as characterization using the markers for immature and mature neurons by using immunocytochemistry and quantitative real-time PCRs.

• Development of a method for differentiating mouse ES cells into neurons.
• Differentiating the mouse ES cells into embryoid bodies prior to induction of neuronal differentiation results in better neuron formation.

## Graphical abstract

**Figure:**
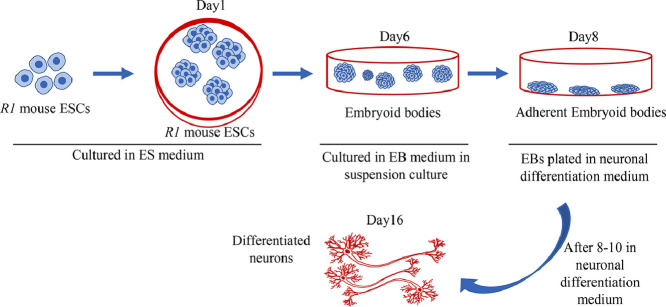

Specifications table

**Table:** 

Subject Area:	Biochemistry, Genetics and Molecular Biology
More specific subject area:	Neurogenetics
Method name:	Reproducible neuronal differentiation from mouse embryonic stem cells
Name and reference of original method:	Ying, Q.-L., Stavridis, M., Griffiths, D., Li, M. & Smith, A. Conversion of embryonic stem cells into neuroectodermal precursors in adherent monoculture. Nat. Biotechnol. **21,** 183-186 (2003).
Resource availability:	Not Applicable

## Method details

### Background

Neurodevelopmental disorders constitute a diverse group of health conditions affecting ≥ 15% of the world population (https://www.who.int/ceh/capacity/neurodevelopmental.pdf). Although the exact cause is not known in many cases, a number of etiological factors have been recognized of which several studies emphasized on genetic factors [1,2]. Multiple family- and population-based studies identified many disease-associated mutations. However, understanding the molecular basis of the neurodevelopmental abnormalities due to these mutations remains as the main challenge. The major reason for this shortcoming is the time-taking and costly procedures involved in developing transgenic mice with the desired mutations. Nevertheless, transgenic mouse models for some of the disease-associated mutations have been developed, characterized and used for testing drugs / small molecules for improving the phenotypes [3], [4], [5] Despite these efforts, many mutations still need to be investigated. Additional challenges include the inability to determine defects in neurodevelopmental stages for mutations that cause embryonic lethality. For example, overexpression of DNMT1 is one of the etiological factors of schizophrenia, but mice overexpressing the protein die around mid-gestation [6]. In the context of these technical and monetary challenges, we earlier proposed that transgenic embryonic stem cells carrying specific mutations are useful and cost-effective for the investigation of the molecular basis of abnormal neurogenesis [7]. Mouse embryonic stem cells (mESCs) have been used for a long time to introduce mutations and generate transgenic mice whereas human embryonic stem cells (hESCs) are being used more recently. Although genetically modified hESCs are closer to the disease conditions than mESCs, the latter have a better recombination rate (homologous as well as non-homologous) for introducing mutations. Further, the availability of rich literature on maintenance and differentiation procedures make mESCs a better choice. Once the basic disease mechanisms are understood using mESCs, hESCs or patient-derived iPSCs or patient tissues can be used for further analyses.

In the context of neurological disorders, successful analysis of the influence of mutations on neurodevelopment hinges upon the ability to reproducibly differentiate neurons from the mESCs. In our work on the analysis of schizophrenia genes in mESCs overexpressing DNMT1, we tried multiple published and publicly available protocols for neuronal differentiation [8]. Unfortunately, these experiments were not very consistent. Therefore, by using a commercially available NDiff 227 neuronal differentiation medium and changing the differentiation conditions, we developed a protocol that consistently resulted in differentiation of mESCs into neurons. Here, we describe a 16-day protocol to obtain neurons from *R1* mESCs and their characterization using quantitative real-time PCRs and immunocytochemistry. The protocol is accompanied by the morphological and molecular features that help in monitoring the progress of neuronal differentiation, possible pitfalls and troubleshooting measures.

## Materials

### Reagents

The lists of reagents, equipment and recipes for making the required solutions are given in Table 1, Table 2, Table 3, Table 4.

**Table 1:** Equipment needed for the procedure described.

Equipment	Company	Catalog #
EVOS-XL Core cell imaging system	ThermoFisher	AMEX1200
Confocal microscope	Leica	TCS SP8
QuantStudio3 Real-time PCR System	ThermoFisher	A28567
NanoDrop^Ⓡ^ 2000	ThermoFisher	ND-2000C
Water bath (37°C)	Sigma-Aldrich	BMSB200012-1EA
CO2 incubator	Eppendorf	CO17111001
Centrifuge	Eppendorf	5702000019
Laminar Air Flow	Clean Air Systems	CBS 1200

**Table 2:** Plasticware needed for the procedure described.

Plasticware	Company	Catalog #
Non-adherent dishes	ThermoFisher	174932
Serological Pipettes	Eppendorf	170354,55,56 N
6-well dishes	Eppendorf	0030720113
Hemocytometer	Sigma-Aldrich	Z359629-1EA
Reusable Bottle Top Filter	Tarsons	520060
0.22µm filtration membrane	Merck-Millipore	GVWP04700
25mm Circular cell culture treated cover slip	HiMedia	TCP-019
Blunt ended forceps	Sigma-Aldrich	Z168726-1EA
15-ml polypropylene conical tubes	Eppendorf	0030122151
250-ml media bottles	Pyrex	CLS1395250
1.5ml centrifuge tubes	Eppendorf	0300 120 086
0.2 ml PCR tubes and caps	ThermoFisher	AB1182

**Table 3:** Reagents needed for the procedure described.

Reagent	Company	Catalog #
Knockout DMEM/F12	ThermoFisher	12660012
ES Grade FBS	HiMedia	RM10435
Glutamax (100X)	ThermoFisher	35050061
Non-Essential Amino Acids (100X)	ThermoFisher	11140050
Penicillin/Streptomycin (100X)	HiMedia	A002A
β-Mercaptoethanol (1000X)	ThermoFisher	21985023
Leukemia Inhibitory Factor (1000X) (10ng/µl)	ThermoFisher	PMC 9484
Trypsin-EDTA	HiMedia	TCL034
Gelatin from porcine skin	Sigma	G1890
Sodium Chloride	Sigma-Aldrich	S7653
Sodium Phosphate dibasic anhydrous	Sigma-Aldrich	S3264
Potassium Phosphate monobasic	Sigma-Aldrich	60220
Potassium Chloride	Sigma-Aldrich	P9541
Glycerol	HiMedia	MB060
4%-Paraformaldehyde	Sigma-Aldrich	100496
TritonX-100	HiMedia	MB031
β-III TUBULIN Rabbit monoclonal antibody (1: 500 dilution)	Cell Signaling Technologies	5568
NESTIN mouse monoclonal antibody (1: 500 dilution)	ThermoFisher	MA1-110
MAP2 Rabbit monoclonal antibody (1: 500 dilution)	Cell Signaling Technologies	8707
OCT-4 Rabbit monoclonal antibody (1: 800 dilution)	Cell Signaling Technologies	83932
GATA6 Polyclonal Antibody (1: 500 dilution)	ThermoFisher	PA1-104
PAX6 Polyclonal Antibody (1: 500 dilution)	ThermoFisher	42-6600
BRACHYURY Polyclonal Antibody (1: 500 dilution)	ThermoFisher	PA5-78422
HISTONE H3 mouse monoclonal antibody (1: 500 dilution)	Cell Signaling Technologies	14269
Anti-mouse IgG Alexa Fluor 555 Conjugate (1: 2000 dilution)	Cell Signaling Technologies	4409
Anti-rabbit IgG Alexa Fluor 488 Conjugate (1:2000 dilution)	Cell Signaling Technologies	4412
Normal Goat serum	HiMedia	RM10701
Bovine Serum Albumin	HiMedia	MB083
DABCO	Sigma-Aldrich	290734
DAPI (4′,6-diamidino-2-phenylindole, dihydrochloride)	ThermoFisher	D1306
Nail Polish	Lakmé or equivalent	-
RNeasy Mini RNA isolation kit	Qiagen	74104
Superscript IV cDNA synthesis kit	ThermoFisher	18091050
2x SYBR Green mix	Bio-Rad	1725121

**Table 4:** Preparation of solutions and culture dishes.

Solutions/Culture Dishes	Procedure
ES Medium (50 ml)	43.4ml Knockout DMEM/F12, 5 ml FBS, 0.5 ml Pen/Strep, 0.5 ml β-Mercaptoethanol (1000X), 0.5 ml Glutamax (100X), 0.5 ml Non-Essential Amino Acids (100X), 0.5 ml Penicillin/Streptomycin (100X) and 50 µl Leukemia Inhibitory Factor (LIF). The contents were mixed and filtered using 0.22µm filtration membrane. The medium was protected from light during storage and handling.
EB Medium (50 ml)	ES medium without LIF was used as EB medium.
Neuronal Differentiation medium	The supplied bottle containing 500 ml NDiff 227 medium was half-thawed in a water bath at 37°C and placed at room temperature in dark till completely thawed. After thawing, the medium was stored as 50ml aliquots in 4°C up to 1 month. Just before use, 0.5 ml Penicillin/Streptomycin (100X) was added to 50ml medium. The medium was protected from light during storage and handling.
Gelatin 0.1% (100 ml)	100 mg gelatin was dissolved in 80 ml of warm (~ 60 °C) distilled water. The volume was made up to 100 ml, sterilized by autoclaving and stored at room temperature.
Gelatin 0.2% (100 ml)	200 mg gelatin was dissolved in 80 ml of warm (~ 60 °C) distilled water. The volume was made up to 100 ml, sterilized by autoclaving and stored at room temperature.
20X PBS (1000 ml)	160g Sodium Chloride (final conc. 2.7 M), 4g Potassium Chloride (final conc. 54 mM), 28.8g Sodium Phosphate (Dibasic; final conc. 200 mM) and 4.8g Potassium Phosphate (Monobasic; final conc. 35.3 mM) were dissolved in 800ml of MilliQ water, pH was adjusted to 7.4 using 1N NaOH or 1N HCl, the volume was made up to 1000ml, sterilized by autoclaving and stored at room temperature.
1X PBS (1000 ml)	50 ml 20X PBS was added to 950 ml MilliQ water, sterilized by autoclaving and stored at room temperature.
Blocking Solution (10 ml)	0.5 ml normal goat serum (from the same species as the secondary antibody; final conc. 5%) was added to 9 ml sterile 1X PBS and mixed well. 30 µl Triton X-100 (final conc. 0.3%) was added and the volume was adjusted to 10 ml. To allow proper mixing, the tube was placed on a rocker at low speed for 30 min. The buffer was freshly prepared for each use.
Antibody Dilution Buffer (10 ml)	30 µl Triton X-100 (final conc. 0.3%)was added to 9 ml 1X PBS and mixed well, followed by addition of 0.1g BSA (final conc. 1%). To allow proper mixing, the tube was placed on a rocker at low speed for 30 min. The volume was adjusted to 10 ml. The buffer was freshly prepared for each use.
Mounting medium (1 ml)	10 µl DABCO (final conc. 1%) and 100 µl sterile 1X PBS were added to 890 µl of glycerol and mixed by pipetting.
DAPI Solution (10 ml)	To make a 1mg/ml DAPI stock solution (final conc. 1µg/ml), 10 mg DAPI was dissolved in 10 mL dimethylformamide (DMF) and stored at -20°C for 6 months. For a working solution (1µg/ml), 10 µl stock solution was dissolved in 10 ml sterile 1X PBS and stored as 1 ml aliquots at -20°C for upto a month.
Culture dishes coated with gelatin	0.1% or 0.2% gelatin was added to cell-culture dishes so that the solution covered the bottom of the dish. The dishes were incubated for 30 min at 37°C. The gelatin was aspirated just before using the plate.
Culture dishes with cover-slips coated with 0.2% Gelatin for immunocytochemistry	0.2% gelatin was added to cell-culture dishes containing cover-slips so that the solution covered the bottom of each dish and the dishes were incubated at room temperature for 5 min. The gelatin was aspirated and the plates were allowed to dry without lid in a laminar air flow in UV light (preferably overnight). Once dried, 0.2% gelatin was added again and incubated for 1 h at 37°C with closed lids. The gelatin was aspirated just before using the plate.

**Procedure**

Culturing of ESCs, generation of embryoid bodies and their subsequent neuronal differentiation was performed as described below.

**A. Culturing and Maintenance of ES cells**

Days 1-3

**Fig. 1:**
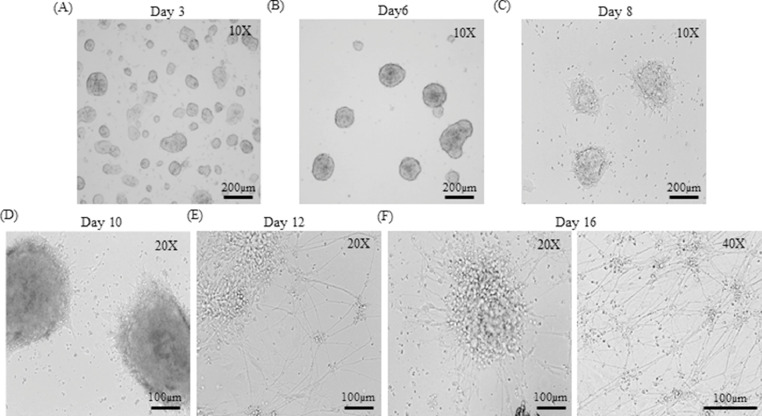
Differentiation of mouse embryonic stem cells (mESCs) into embryoid bodies and neurons. Light microscope images of (A) Colonies of *R1* mESCs cultured for three days. (B) Embryoid bodies (EBs) in suspension culture in EB medium (Day 6). (C) EBs attached onto gelatin coated dishes in differentiation medium (Day 8). (D) Differentiating cells showing small projections in neuronal differentiation medium (Day 10). (E) Differentiating cells with rosette formation and appearance of neurites (Day 12). (F) Differentiated neurons (Day 16) at lower magnification (10X) and higher magnification (40X).

1. Gelatin-coated dishes were prepared as described in Table 4. Cryopreserved *R1* ES cell line was removed from liquid nitrogen and quickly swirled in a water bath at 37°C until half-thawed. The vial was wiped thoroughly with 70% ethanol and its contents were emptied in 5ml of warm ES medium in a laminar air flow. The cells were then centrifuged at 1,000 rpm for 5 min. The medium containing cryoprotectant was aspirated without disturbing the cell pellet. The pellet was resuspended in 1 ml of ES medium. For counting viable cells, 10 µl of cell suspension was mixed with 140 µl of 1X PBS and 50 µl of 0.4% Trypan Blue stain and incubated for 5 min at room temperature. 10 µl of cell suspension was then applied onto a hemocytometer, covered using a glass cover slip and observed under a microscope to count the viable cells. Viable cells have a clear cytoplasm whereas nonviable cells have a blue cytoplasm. Cell counting was performed to estimate the number of viable cells as described by Strober et al. (2001) [9].
2. Gelatin was aspirated and the ES cells were seeded at a density of ~50,000 cells/ cm^2^ in 2 ml ES medium into 6-well dishes. The cells were incubated in a CO_2_ incubator at 37°C.
3. On day 2, ~90% of the cells were attached. The cells were replaced with fresh medium every day.
4. By day 3, the ESCs started growing as colonies (Fig. 1A).

**B. Generation of Embryoid Bodies**

Day 4

5. By day 4 the colonies had grown in size and covered most of the culture area (~80-90% confluence). The medium was replaced with 2 ml of IX PBS (Table 4) and washed by gentle swirling. The wash was repeated one more time.
6. 0.5 ml of 1X Trypsin-EDTA (Table 4) was added to the cells and incubated at 37°C for 5 min. The trypsinization was monitored under an inverted microscope and the cells were thoroughly pipetted up and down using a 1 ml pipette tip. Another 0.5 ml of 1X Trypsin-EDTA was added and the incubation was continued for another 5 min at 37°C. The cells were dissociated by pipetting again to obtain a single cell suspension. 1 ml of ES medium was added to inactivate Trypsin.
7. The cell suspension was transferred to a 15 ml centrifuge tube and centrifuged at 1,000 rpm for 5min. Without disturbing the pellet, the medium was aspirated.
8. The cell pellet was resuspended in 1 ml of ES medium and counted using a hemocytometer as described in Step1. For further culturing, the cells were seeded onto a gelatin-coated T-25 or T-75 flask(s).
9. For generation of embryoid bodies (EBs), ~50,000 ES cells/cm^2^ were plated in 2 ml of EB medium (Table 4) onto non-adherent culture dishes. Care was taken that the pipette tip did not scratch the well surface as it would lead to attachment.

Day 5

10. On day 5, EBs had formed after overnight incubation and appeared as spherical aggregates of cells. The EBs were cultured for another day (day 6) in EB medium (Fig. 1B).
11. Six-well dishes with or without coverslips were coated with 0.2% gelatin as described above.

**C. Neuronal Differentiation**

Days 6-7

12. The EBs were collected in suspension from the culture dish, transferred to a 15 ml centrifuge tube and left undisturbed for 15 min to allow the EBs to settle down.
13. The medium was aspirated without disturbing the EBs.
14. The settled EBs were resuspended in 1 ml of neuronal differentiation medium.
15. The EBs were plated at a density of ~5 EBs per/cm^2^ in 2ml of neuronal differentiation medium onto 0.2% gelatin coated dishes.
16. The EBs were uniformly spread over the culture area by rocking the plate in horizontal and vertical motions. Swirling of the EBs was avoided as it would result in settling of all the EBs in the middle of the well. Larger dishes could not allow uniform spreading of the EBs and were therefore avoided.
17. The EBs, once evenly spread on the culture surface, were gently placed in the CO_2_ incubator and were left undisturbed for 48 h.

Day 8

18. On day 8, most of the EBs were attached (Fig. 1C). The medium was removed along with any unattached EBs and 3ml of fresh medium was added. After this point, half of the medium was replenished every day. Care was taken that at no point, the differentiating cells were disturbed or exposed to air and light.

Days 9-12

19. Half of the medium was continued to be replenished every day without disturbing the differentiating cells.
20. From day 9 onwards, cell death concomitant with neural differentiation was observed.
21. From day 10 onwards, small projections were observed (Fig. 1D).
22. On day 12, rosette-like formation of cells was observed with elongated projections resembling neurites (Fig. 1E).

Day 13-16

23. By days 15-16, most of the cells differentiated with extensive neurite formation and branching (Fig. 1F). The neurons were then used for immunocytochemistry or other downstream experiments.

## Method validation

**A. Monitoring differentiation by quantitative real-time PCRs**

**Procedure**

1. RNA samples were prepared from different cells using the RNeasy kit as per manufacturer's instructions and quantified using NanoDrop^Ⓡ^.
2. 5 µg each of the RNA samples were converted to cDNA using Superscript IV cDNA synthesis kit as per manufacturer's instructions.
3. ~50 ng each of the cDNA samples were then mixed with gene-specific primer mix (0.2 µM each) (Table 5), 2x SYBR Green mix and PCR grade water to a final volume of 10 µl in 0.2 ml PCR tubes.

**Table 5:** Oligonucleotide sequences used for real-time PCR analysis.

Gene Name	Forward Primer	Reverse Primer	Annealing Temperature	Size (bp)
*Brachyury*	GCCAAAGAAAGAAACGACCA	ATTGTCCGCATAGGTTGGAG	60°C	264
*Gapdh*	GAAGGGCTCATGACCACAGTCC	TCATACTTGGCAGGTTTCTCCAGGC	60°C	256
*Gata6*	GTGCAATGCATGCGGTCTCTACAG	TTCATAGCAAGTGGTCGAGGCACC	60°C	222
*Gfap*	CACGAACGAGTCCCTAGAGC	ATGGTGATGCGGTTTTCTTC	60°C	234
*Itgam*	CTCATGGTCACCTCCTGCTT	CAAGCTTGTATAGGCCAGCA	60°C	406
*Map2*	AGCCGCAACGCCAATGGATT	TTTGTTCTGAGGCTGGCGAT	60°C	313
*Mbp*	CTTCAAAGACAGGCCCTCAG	GCTGTCTCTTCCTCCCTTCC	60°C	213
*Nanog*	CTGAGCTATAAGCAGGTTAAGACC	TGCTGAGCCCTTCTGAATC	60°C	114
*Nestin*	GAAGTGGCTACATACAGGACTC	GGTCAGGAAAGCCAAGAGAA	60°C	94
*Oct4*	CTCCCGAGGAGTCCCAGGACAT	GAGAACCTTCAGGAGATATGCAAATCGG	60°C	179
*Pax6*	AACGCGAGGAAGATGTGTCT	GGTACGTCTGTGTGCCTGAC	60°C	303
*β III Tubulin*	TCAGCGATGAGCACGGCATA	CACTCTTTCCGCACGACATC	60°C	301
4. The reactions were used for real-time PCR analyses (PCR conditions: 1 cycle of 95°C: 2 min; 40 cycles of 95°C: 30 sec, 60°C: 30 sec, 72°C: 30 sec; 1 cycle of 72°C: 1 min). *Gapdh* was used as endogenous reference control and fold-changes were calculated by the 2^−ΔΔCt^ method [10].

All the experiments were done with at least three biological and two technical replicates. The expression levels of the different transcripts from EBs and neurons were compared with those from ESCs by the student's two-tailed t-test and *p* <0.05 was taken as statistically significant.

## Results

**Fig. 2:**
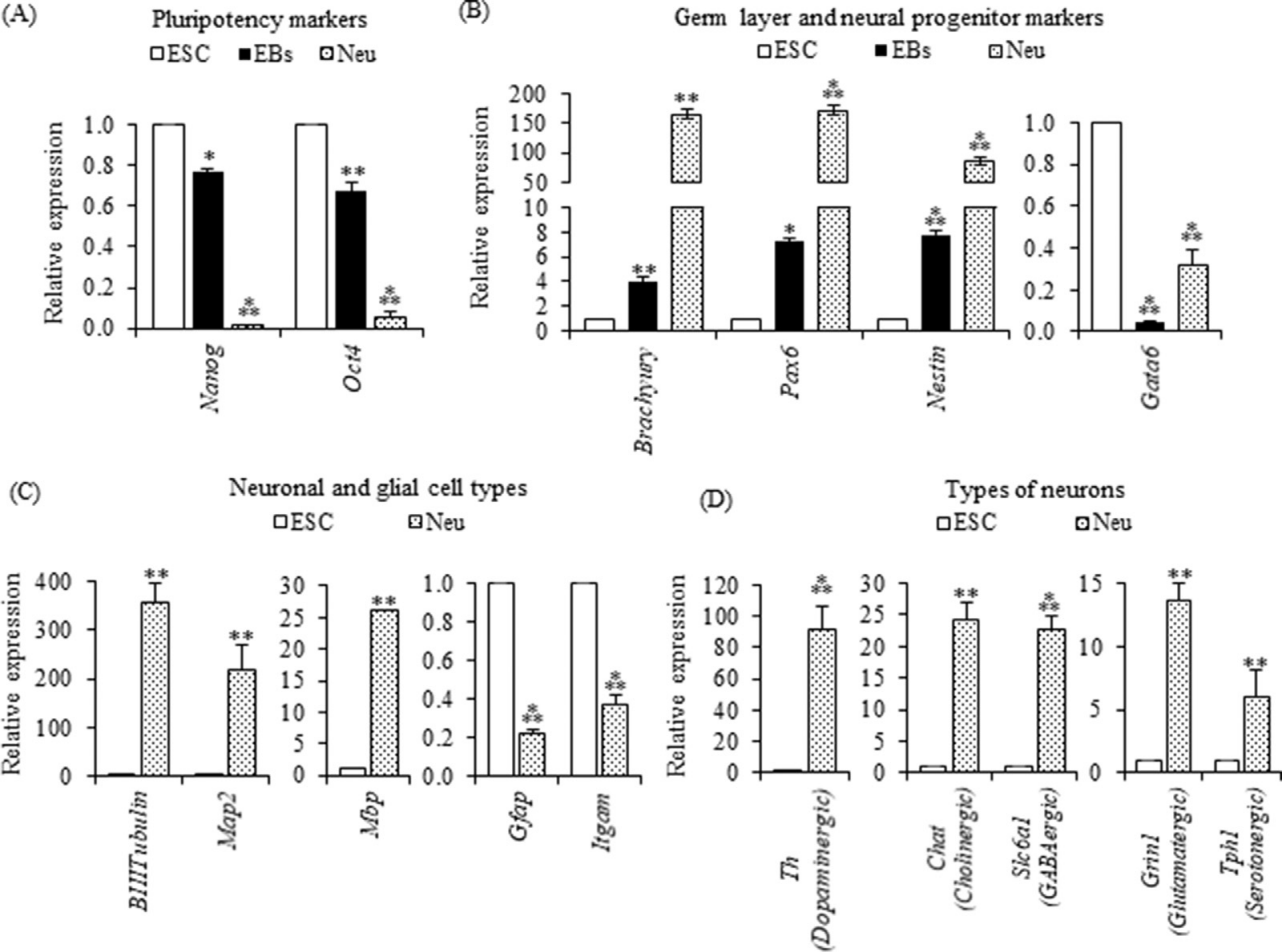
Monitoring differentiation by Real-time PCR analysis. Analysis of transcript levels of (A) Pluripotency markers: *Oct4* and *Nanog.* (B) Germ layer markers: *Brachyury* (Mesoderm), *Gata6* (Endoderm), *Pax6* (Ectoderm) and neural progenitor marker *Nestin*. (C) Neuronal and glial markers: *β-III Tubulin* (Immature neurons), *Map2* (Mature neurons), *Mbp* (Oligodendrocytes), *Gfap* (Astrocytes) and *Itgam* (Microglia). (D) Markers for different types of neurons produced: *Th*: Tyrosine hydroxylase (Dopaminergic neurons), *Chat*: Choline acetyltransferase (Cholinergic neurons), *Slc6a1*: Solute carrier family 6 member 1 (GABAergic neurons), *Grin1*: Glutamate ionotropic receptor NMDA type subunit 1 (Glutamatergic neurons), *Tph1*: Tryptophan hydroxylase 1 (Serotonergic neurons). *, ** and *** indicate *p*-values < 0.05, < 0.01 and < 0.001, respectively.

In order to monitor the process of differentiation of ESCs into embryoid bodies and neurons, we studied the transcript levels of markers specific for pluripotency, germ layers, neuronal and glial cells. As expected, the transcript levels of pluripotency markers (*Oct4* and *Nanog*) were significantly downregulated during the differentiation into EBs as well as neurons (Fig. 2A). Analysis of transcript levels of germ layer markers revealed significantly increased levels of *Brachyury* (mesoderm marker) and *Pax6* (ectoderm marker) in EBs and neurons (Fig. 2B). However, *Gata6* (endoderm marker) was observed to be decreased in both EBs and neurons. Importantly, we also observed increased transcript levels of *Nestin* (neural progenitor marker) in EBs and neurons (Fig. 2B). These results indicated that the differentiation method used here resulted in a neural commitment of pluripotent cells.

Analysis of transcript levels of neuronal markers showed significantly increased levels of *β-III Tubulin* (immature neurons) and *Map2* (mature neurons). Among the glial cell markers, *Mbp* (oligodendrocytes) showed significantly increased transcript levels in neurons whereas *Gfap* (astrocytes) and *Itgam* (microglia) were downregulated. These results suggested that this method of differentiation resulted in an efficient neuronal differentiation into immature and mature neurons.

Analysis of transcript levels of markers for different neurons indicated the presence of dopaminergic, cholinergic, GABAergic, glutamatergic and serotonergic neurons upon differentiation. As expected, these markers expressed at significantly higher levels in neurons than ESCs. Data on three biological replicates showed a batch to batch variation with a standard error of ~12.1 % (Fig. 2D). These results suggested that the differentiation protocol used here was more or less reproducible for obtaining neurons from mouse ESCs.

**B. Immunocytochemistry**

**Procedure**

1. Cells plated on coverslips were used for these experiments. When the cells are ready for the experiments, the medium was aspirated and the cells were washed thrice with 2 ml of warm 1X PBS (added to the walls of the well) by gently rocking the dish manually.
2. The cells were fixed with 1ml of 4% paraformaldehyde for 15 min at room temperature.
3. The fixative was aspirated and the cells were again washed thrice with 2 ml of 1X PBS for 5 min each time at room temperature. The washes were performed by placing the dishes on a rocker at low speed.
4. The cells were blocked in 1 ml of blocking buffer for 1 h at room temperature by placing the dishes on a rocker at low speed.
5. During the blocking step, the primary antibodies were diluted in 0.5 ml of antibody dilution buffer, as per the dilutions given above. The diluted antibodies were mixed properly for at least 15 min by placing the tube on a rocker at low speed.
6. After one hour, the blocking buffer was replaced with diluted primary antibodies and incubated overnight at 4°C by placing the dishes on a rocker at low speed.
7. After incubation, the antibody solution was removed and the cells were washed thrice with 1X PBS as described above. The cells were then incubated with 1 ml of diluted fluorochrome-conjugated secondary antibodies for two hours at room temperature in dark by placing on a rocker at low speed.
8. The cells were washed thrice with 1X PBS as described above and counterstained with 1 ml of DAPI solution for 5 min at room temperature in dark.
9. The DAPI solution was aspirated and the cells were washed thrice with 1X PBS as described above and used for mounting.
10. 10-20 µl of mounting medium was placed in the middle of a clean and dry glass slide. The coverslips containing stained cells were then picked up gently using sterile forceps and placed on the mounting medium, avoiding any air bubbles. The edges of the coverslips were sealed using a transparent nail polish.
11. The slides were left undisturbed for 3 to 4 h at room temperature in dark, before using them for confocal microscopy. For long-term storage, the slides were stored at 4°C in dark and in flat position.
12. Images were captured and analyzed using LasX Life science software platform (Leica).

Note: Immunocytochemistry experiments with ~50 EBs each were performed in 1.5 ml transparent centrifuge tubes in 200 µl volumes. For each step, mixing was done by gentle pipetting followed by centrifugation at 1000 rpm for 30 s. At the end of all steps, the EBs were mixed in 20 µl of mounting medium, covered with coverslips and processed as described above.

## Results

**Fig. 3:**
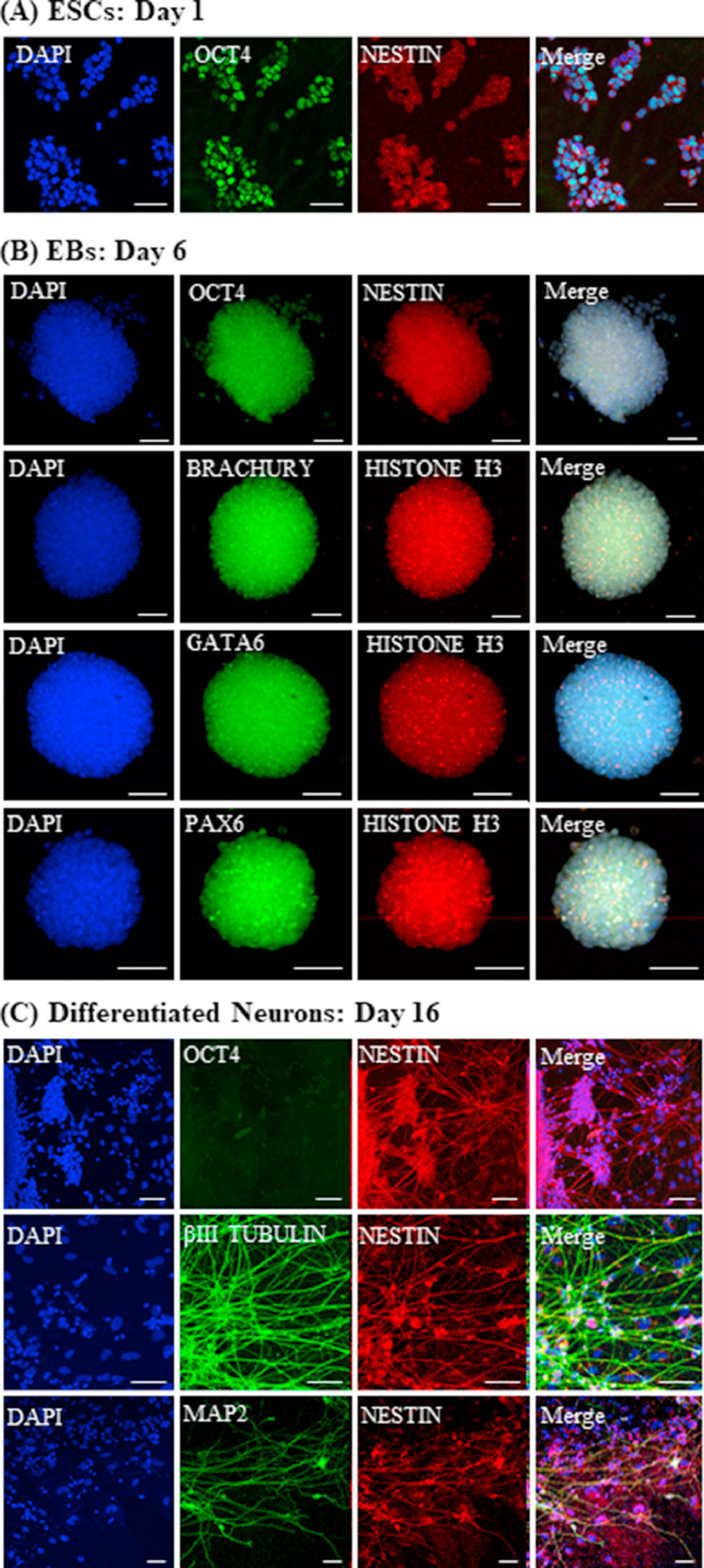
Immunocytochemistry of ESCs, EBs and neurons. Confocal microscopy images of (A) ESCs with antibodies to OCT4 (green, pluripotency) and NESTIN (red, neural progenitor). (B) EBs with antibodies to OCT4 (green), NESTIN (red), BRACHYURY (green, mesoderm), GATA6 (green, endoderm), PAX6 (green, ectoderm) and HISTONE H3 (red). Scale: 50 µm. (C) Differentiated neurons with OCT4 (green), NESTIN (red), β-III TUBULIN (green, immature neurons) and MAP2 (green, mature neurons). In all experiments DAPI (blue) was used to stain the nuclei. (For interpretation of the references to color in this figure legend, the reader is referred to the web version of this article.)

We studied the expression of pluripotency (OCT4), germ layer (BRACHYURY, PAX6 and GATA6), neural progenitor (NESTIN) and neuronal markers (β-III TUBULIN and MAP2) by immunocytochemistry of the relevant cell types (Fig. 3). OCT4 levels were observed to be lower in EBs than ESCs, whereas in case of neurons, almost no OCT4 expression was seen. The EBs at two days of differentiation showed expression of all three germ layer markers (Fig. 3B). NESTIN expression was observed to be higher in EBs and neurons than ESCs. The neurons were also well branched and positive for β-III TUBULIN and MAP2 (Fig. 3C). Although we detected the presence of mRNA for non-neuronal cells (oligodendrocytes, astrocytes and microglia) but there was no detectable signal by immunocytochemistry.

These results suggest that the method of differentiation described here results in efficient and reproducible differentiation of pluripotent ESCs into mature neurons in about 16 days.

**Troubleshooting:**

**Table:** 

**Problem**	**Suggestion**
String-like aggregation of cells after overnight incubation of ESCs in EB medium	Gentle pipetting to break the strings with a 1ml pipette tip, ~8-10 times resolved the problem and spherical EBs were obtained on the next day.
Unattached EBs after 48 hours of incubation in neuronal differentiation medium	Increased the incubation time to a maximum of 72 h. If attached after 72 h, differentiation was continued as described. However, if EBs remain unattached after 72 h, the process was repeated using fresh 0.2% Gelatin coated dishes.
Absence of neuronal differentiation	Overcrowding of differentiating cells resulted in inhibition of differentiation as optimal seeding density plays an important role. Reduction in the seeding density of EBs, resulted in improved neuronal differentiation.

## Declaration of Competing Interest

The authors declare that they have no known competing financial interests or personal relationships that could have appeared to influence the work reported in this paper.
